# Restless legs syndrome and cardiovascular diseases: A case-control study

**DOI:** 10.1371/journal.pone.0176552

**Published:** 2017-04-26

**Authors:** Marion Cholley-Roulleau, Sofiene Chenini, Séverine Béziat, Lily Guiraud, Isabelle Jaussent, Yves Dauvilliers

**Affiliations:** 1 Unité des Troubles du Sommeil, Neurologie, Hôpital Gui-de-Chauliac, Montpellier, France; 2 Inserm, U1061, Montpellier, France; 3 Université Montpellier, Montpellier, France; Associazione OASI Maria SS, ITALY

## Abstract

**Objective:**

The association between restless legs syndrome (RLS), cardiovascular diseases (CVD) and hypertension is inconsistent. This case-control study examined i) the association between primary RLS, CVD and hypertension by taking into account many potential confounders and ii) the influence of RLS duration, severity and treatment, sleep and depressive symptoms on CVD and hypertension in primary RLS.

**Methods:**

A standardized questionnaire to assess the RLS phenotype, history of CVD and hypertension, sleep and depressive symptoms, drug intake and demographic/clinical features was sent to the France-Ekbom Patients Association members. A CVD event was defined as a self-reported history of coronary heart disease, heart failure, arrhythmia or stroke. Hypertension was also self-assessed. Current treatment for hypertension and arrhythmia also defined underlying hypertension and arrhythmia. Controls without RLS and without consanguinity were chosen by the patients.

**Results:**

487 patients with primary RLS (median age 71 years; 67.4% women) and 354 controls (68 years, 47.7% women) were included. Most of the patients (91.7%) were treated for RLS, especially with dopaminergic agonists. The median age of RLS onset was 45 years. CVD and hypertension were associated with RLS in unadjusted association, but not after adjustment for age, sex and body mass index. Patients with RLS and with CVD and/or hypertension were significantly older, with hypercholesterolemia, sleep apnea and older age at RLS and at daily RLS onset compared with patients without CVD and/or hypertension. No significant difference was found for other RLS features, ferritin levels, daytime sleepiness, insomnia and depressive symptoms.

**Conclusion:**

Despite some limitations in the design of this study, we found that most of the treated patients for primary RLS had no association with CVD and hypertension after controlling for key potential confounders. Comorbid CVD or hypertension was associated with cardiovascular risk factors, but not with RLS features except for older age at onset.

## Introduction

Restless legs syndrome (RLS), also known as Willis-Ekbom disease, is a common neurological sensorimotor disorder that often impairs sleep and quality of life [1,2]. RLS is frequently comorbid with diseases associated with increased cardiovascular risk such as obesity, hypercholesterolemia, diabetes mellitus, obstructive sleep apnea, insomnia, or depression [3,4–6]. RLS is often classified as primary/idiopathic or secondary/symptomatic when it occurred with associated diseases such as iron deficiency anemia, chronic renal failure, hemochromatosis, Crohn’s and coeliac diseases, arthritis, neurological disorders (Parkinson’s disease, polyneuropathy, multiple sclerosis), diabetes, and medications such as neuroleptics [1,2].Periodic limb movements (PLMs) during sleep are present in 80% of patients with RLS [7]. PLMS are often associated with micro-arousals that contribute to sleep fragmentation and repeated increases of blood pressure and heart rate throughout the night [8] that represent an increased risk for hypertension [9] and cardiovascular diseases (CVD) [10]. Based on its pathophysiology (i.e. a strong genetic predisposition and dysregulation of iron metabolism and the dopaminergic system), RLS *per se* may also contribute to sympathetic hyperactivity and then to hypertension and CVD [11].

However, the association between RLS and CVD or hypertension remains unclear in the literature [10,12–21] with several differences in study design, sample size, adjustments for potential confounders and heterogeneity in the assessment of RLS (diagnosis, duration, frequency, severity and presence of potential comorbidities that could suggest secondary RLS). A recent and well-designed large retrospective cohort study reported that primary RLS is associated with higher risk of hypertension but not of incident CVD, whereas secondary RLS is associated with an increased risk for both [18]. However, to the best of our knowledge, no study has examined the effect of daytime sleepiness, insomnia complaints and depressive symptoms on the association between RLS and 1) CVD 2) hypertension. Indeed, sleep disruption, insomnia, daytime sleepiness and depression are frequent comorbid conditions in RLS [22,23].and they might increase the risk of CVD and hypertension [24,25,26]. The association between RLS, CVD and hypertension has not been thoroughly assessed in clinical populations, especially in patients treated for RLS.

The aims of the present case-control study were: i) to examine the association between primary RLS, CVD and hypertension by taking into account several potential confounders, including demographic, lifestyle and health (e.g., depression and chronic disorders) variables and sleep complaints; and ii) to study the effect of RLS duration and severity as well as of sleep and depressive symptoms on CVD and hypertension.

## Methods

### Study population

Patients with primary RLS were recruited in 2013 among volunteers of the 2700 adult members of the France-Ekbom Patients Association (AFE) dedicated to patients with RLS. Information on the study objectives was sent to each AFE member with a valid postal address, together with two anonymous questionnaires that included several clinical assessment scales (see below): one to be completed by the patient and one by a control subject chosen by the patients among their entourage (e.g., spouse/partner, friends or colleagues). Controls were without RLS, did not take RLS-related drugs and had no consanguinity with the patient.

Completed questionnaires were sent back to AFE where data were uploaded in a dedicated database. The subjects consented to participate in the study by answering and sending back the questionnaires. All participants agreed to take part in this research program, and gave their written informed consent for the study that was approved by the local ethics committees (Comité de Protection des Personnes–Sud Méditérranée).

### RLS assessment

RLS was defined using the International RLS Study Group (IRLSSG) criteria [1]. Standardized questions addressed the presence of the four minimal diagnostic criteria of the IRLSSG at time of study or before starting medication for treated patients: 1/ Do you feel or have you ever felt an irresistible urge to move your legs? 2/ If you feel or you have ever felt an irresistible urge to move your legs, does it begin or become worse during periods of rest or inactivity, such as sitting or lying down? 3/ If you feel or you have ever felt an irresistible urge to move your legs, does it get better, at least partially, by movements such as walking or stretching your legs? 4/ If you feel or you have ever felt an irresistible urge to move your legs, does-it begin or become worse during the evening or the night? A positive answer to all four questions was required for a presumed diagnosis of RLS [1] and inclusion in the study.

Patients with RLS secondary to pregnancy, chronic renal failure, hemochromatosis or neurological diseases (Parkinson’s disease, multiple sclerosis, polyneuropathy, fibromyalgia, dementia, myelitis, spin cerebellar ataxia and narcolepsy) were excluded (see below).

Controls having at least one positive answer among the four cardinal RLS questions or any of these above diseases were excluded for this study.

Patients were asked to record the age of RLS onset, age of daily RLS onset, last ferritin measurement, presence of RLS symptoms in the arms, family history of RLS, and current RLS treatment [dopaminergic agonists (ropinirole, pramipexole, rotigotine), alpha 2 delta-ligands (pregabaline, gabapentin), levodopa-benserazide, clonazepam and opioids (codeine, tramadol, oxycodone)]. They also filled in the IRLSSG severity scale [27] and the RLS quality of life scale [28]. RLS augmentation occurring after the initiating treatment was defined by the presence of two or three (probable) or of four or five (certain) of the following symptoms compared to the untreated period: increase in symptom severity, several hours of advancement of symptoms during the day, spreading of symptoms to other body parts, shorter latency to symptoms when at rest and shorter duration of relief after treatment [29].

### CVD and hypertension

Data on the history of vascular diseases diagnosed by a doctor were completed by all participants through a standardized questionnaire. A CVD event was defined as a self-reported history of coronary heart disease (e.g., angina pectoris, myocardial infarction with or without revascularization process), chronic heart failure, arrhythmia (e.g. atrial arrhythmia, junctional arrhythmia, ventricular arrhythmia and heart block) or stroke. In addition hypertension was assessed using the self-administered questionnaire. Age at CVD onset, family history of CVD and related medication intake were also recorded in the questionnaire. Current treatment for hypertension (including antihypertensive, diuretic, peripheral dilators, beta blocking agents, calcium channel blockers and agents acting on the renin-angiotensin system) and antiarrhythmic treatment (antiarrhythmic class I and III) were coded according to the World Health Organization’s Anatomical Therapeutic Chemical (ATC) Classification that also defined the presence of hypertension and arrhythmia.

### Socio-demographic, clinical and sleep variables

A similar questionnaire completed by all participants included questions on demographic characteristics, level of education, marital status (living in couple or not), daily life habits such as alcohol consumption, smoking status and physical activity and anthropometric data, including height and weight to calculate the body mass index (BMI).

Data on personal and family histories of chronic disorders (e.g., diabetes, hyperlipidemia, neurological and renal diseases, sleep apnea syndrome) and pregnancy, and the age at onset were self-rated by all participants through the questionnaire. All medications taken during the previous month were detailed together with their dose and age at intake onset. Subjects receiving antidiabetic and cholesterol-lowering medications were classified as having these conditions.

Subjects also filled in the Beck Depression Inventory [30], a 21-item self-assessment tool (scores between 14 and 19 indicated mild depression and between 20 and 69 moderate to severe depression), the Epworth Sleepiness Scale (ESS) to evaluate daytime sleepiness (EDS) (total score >10: EDS) [31] and the Insomnia Severity Index (ISI), a 7-item self-report scale on subjective insomnia symptoms [32] (cut-off score >14: significant insomnia and >20: severe insomnia).

### Statistical analysis

Categorical variables were presented as number with percentages and quantitative variables as medians with ranges. Patients with RLS and controls were compared using logistic regression models. Associations were quantified with odds-ratios (OR) and their 95% confidence intervals (CI). Demographic and clinical variables associated with RLS in univariate analyses (with p<0.15) were included in logistic regression models to estimate the adjusted OR and their 95% CI for the associations between CVD, hypertension and RLS. Logistic regression models were also used to evaluate the relationships between the patients’ clinical and social characteristics and CVD and hypertension. When appropriate, the interaction terms were tested using the Wald-χ2 test given by the logistic regression model. Significance level was set at p<0.05. Analyses were performed using SAS (version 9.4; SAS Inc., Cary, North Carolina).

## Results

### Study population

A total of 905 questionnaires (524 patients, 381 controls) were returned to AFE within six months. Thirty-seven patients (incomplete questionnaire: n = 5; no consent for electronic data storage: n = 1; absence of the four cardinal RLS features: n = 10; secondary RLS: n = 21) and 27 controls (incomplete questionnaire: n = 3; no consent; n = 1; consanguinity: n = 1; dopaminergic/alpha 2 delta ligand intake: n = 22) were excluded. Therefore, analyses were performed on a population of 487 patients with primary RLS (median age: 71 years, range 26 to 95 years, 67.4% women) and 354 controls (median age: 68 years, range 27 to 92 years, 47.7% women).

Among patients, the median age of RLS onset was 45 years and the median symptom duration was 14 years (Table 1). At study inclusion, 91.2% of patients were taking an RLS-related drug: 83.8% a dopaminergic agonist, 14.0% alpha 2 delta ligands and 1.6% levodopa. RLS was moderate in 18.9%, severe in 58.6% and very severe in 21.6% of patients. A family RLS history was reported by 46.5% of patients. A low ferritin level (<50ng/ml) was found in 9.3% of patients. A probable RLS augmentation was reported by 30.6% of patients and almost certain augmentation by 9.5%.

**Table 1:** Characteristics of patients with primary restless legs syndrome (RLS).

	N = 487	
Variables	n	%
RLS severity (IRLSSG^(1)^ scale)		
[0–10] mild	4	0.90
[11–20] moderate	84	18.88
[21–30] severe	261	58.65
[31–40] very severe	96	21.57
RLS family history (Yes)	219	46.50
Age of RLS symptom onset, in years^(2)^	481; 45 [2–84]	
Duration of RLS symptoms ^(2)^	481; 24 [0–86]	
Age of daily RLS symptom onset, in years^(2)^	451; 54 [2–90]	
Duration of daily RLS symptoms ^(2)^	451; 14 [0–72]	
Ferritin, ng/ml (<50)	17	9.34
Treatment for RLS (Yes)	444	91.17
RLS augmentation		
No	259	59.95
Probable	132	30.56
Certain	41	9.49
RLS upper limb (Yes)	225	47.47
RLS Quality of life–global score^(2)^	442; 33 [3–60]	

^(1)^ International Restless Legs Syndrome Study Group

^(2)^ Quantitative variables are expressed as number; median [minimum value–maximum value]

Compared with controls, patients with primary RLS were older, more frequently women, overweight or obese, reported more frequently sleep apnea syndrome, depressive symptoms, insomnia and EDS symptoms (p<0.05 for all comparisons) (Table 2). Patients with RLS also consumed significantly more antidepressant drugs, but less alcohol. Therefore, the subsequent analyses were adjusted for these factors. No significant difference between groups was found concerning other cardiovascular risk factors (smoking status, physical activity, dyslipidemia, diabetes or family history of CVD).

**Table 2:** Demographic and clinical characteristics of patients with primary restless legs syndrome (RLS) and controls.

	Controls N = 354		Primary RLS N = 487			
	n	%	n	%	OR [95% CI]	Global p
Sex (Women)	169	47.74	328	67.35	2.26 [1.70;2.99]	<0.0001
Age, years						
<61	113	31.92	97	19.92	1	<0.0001
[61–69[	90	25.42	120	24.64	1.55 [1.06;2.28]	
[69–77[	85	24.01	125	25.67	1.71 [1.16;2.52]	
≥77	66	18.64	145	29.77	2.56 [1.72;3.81]	
Smoker status						
Non-smoker	235	66.76	336	69.28	1	0.71
Current smoker	25	7.10	34	7.01	0.95 [0.55;1.64]	
Former smoker	92	26.14	115	23.71	0.87 [0.63;1.20]	
Alcohol, g/day						
No drink	84	24.28	140	29.66	1	0.03
≤12	172	49.71	248	52.54	0.87 [0.62;1.21]	
]12–24]	61	17.63	48	10.17	0.47 [0.30;0.75]	
]24–36]	14	4.05	18	3.81	0.77 [0.36;1.63]	
>36	15	4.34	18	3.81	0.72 [0.34;1.50]	
Physical Activity						
No	102	29.14	118	24.38	1	0.19
Yes, moderate	219	62.57	332	68.60	1.31 [0.96;1.80]	
Yes, intense	29	8.29	34	7.02	1.01 [0.58;1.78]	
Body Mass Index, kg/m^2^						
<25	198	57.56	232	48.23	1	0.0002
[25–30]	125	36.34	176	36.59	1.20 [0.89;1.62]	
>30	21	6.10	73	15.18	2.97 [1.76;5.00]	
Family history of cardiovascular diseases (Yes)	100	28.65	158	33.19	1.24 [0.92;1.67]	0.16
Hypercholesterolemia (Yes)	101	28.77	118	24.79	0.82 [0.60;1.11]	0.20
Diabetes mellitus (Yes)	28	7.93	30	6.17	0.76 [0.45;1.30]	0.32
Sleep apnea syndrome (Yes)	21	6.18	75	16.59	3.02 [1.82;5.01]	<0.0001
Antidepressant intake (Yes)	31	8.91	67	13.96	1.66 [1.06;2.60]	0.03
Beck Depression Inventory						
No depression	224	70.44	199	44.62	1	<0.0001
Mild depression	59	18.55	134	30.04	2.56 [1.78;3.67]	
Moderate depression	21	6.60	74	16.59	3.97 [2.36;6.68]	
Severe depression	14	4.40	39	8.74	3.14 [1.65;5.95]	
Epworth Sleepiness Scale (≥11)	52	16.20	206	45.47	4.31 [3.04;6.12]	<0.0001
Insomnia severity index						
No insomnia	185	55.56	35	7.51	1	<0.0001
Mild insomnia	111	33.33	140	30.04	6.67 [4.30;10.3]	
Moderate insomnia	31	9.31	228	48.93	38.88 [23.1;65.4]	
Severe Insomnia	6	1.80	63	13.52	55.50 [22.3; 138]	

### Association between RLS and CVD or hypertension

CVD was associated with RLS in unadjusted association (OR = 1.58 95% CI = 1.10–2.27, p = 0.01), but not after adjustment for age, sex and BMI (Model 1,OR = 1.47 95% CI = 0.99–2.19, p = 0.06) or other potential confounders (alcohol consumption, anti-depressant intake, sleep apnea syndrome, depression, insomnia and EDS) (Model 2, OR = 1.19 95%CI = 0.63–2.24, p = 0.59) (Table 3). Whatever the model, no significant association was found between RLS and coronary heart diseases, chronic heart failure, arrhythmia or stroke before and after adjustment for hypertension. Similarly, hypertension was associated with RLS in the univariate analysis (OR = 1.63 95% CI = 1.22–2.19], but not after adjustment for confounding variables (Model 1, p = 0.14; Model 2, p = 0.86). No significant interactions were found between 1) CVD 2) hypertension and gender, age, or BMI in association with the presence of RLS.

**Table 3:** Cardiovascular diseases and hypertension in patients with primary restless legs syndrome (RLS) and controls.

	Controls N = 354		Primary RLS N = 487		model 0		model 1		model 2	
Variable	n	%	n	%	OR [95% CI]	p	OR [95% CI]	p	OR [95% CI]	p
**CARDIOVASCULAR DISEASES**^(1)^										
No	297	84.86	377	78.05	1		1		1	
Yes	53	15.14	106	21.95	1.58 [1.10;2.27]	0.01	1.47 [0.99;2.19]	0.06	1.19 [0.63;2.24]	0.59
Arrhythmia										
No	314	89.20	418	86.54	1		1		1	
Yes	38	10.80	65	13.46	1.28 [0.84;1.97]	0.25	1.04 [0.65;1.65]	0.88	0.93 [0.45;1.94]	0.85
Chronic heart failure										
No	339	97.69	461	95.64	1		1		1	
Yes	8	2.31	21	4.36	1.93 [0.84;4.41]	0.12	1.64 [0.69;3.89]	0.26	0.71 [0.14;3.74]	0.69
Coronary heart diseases										
No	340	96.87	465	95.48	1		1		1	
Yes	11	3.13	22	4.52	1.46 [0.70;3.06]	0.31	2.10 [0.95;4.66]	0.07	1.60 [0.46;5.51]	0.46
Stroke										
No	342	97.16	464	95.28	1		1		1	
Yes	10	2.84	23	4.72	1.69 [0.80;3.61]	0.17	1.51 [0.68;3.37]	0.31	1.40 [0.39;5.04]	0.61
**HYPERTENSION**										
No	245	69.80	283	58.59	1		1		1	
Yes	106	30.20	200	41.41	1.63 [1.22;2.19]	0.001	1.28 [0.92;1.78]	0.14	0.96 [0.58;1.58]	0.86

^(1)^ Cardiovascular diseases: arrhythmia, chronic heart failure, coronary heart diseases (e.g., angina pectoris, myocardial infarction), stroke

Model 0: unadjusted associations

Model 1: adjusted for age, sex, BMI

Model 2: adjusted for all covariates of model 1 plus alcohol consumption, anti-depressant intake, sleep apnea, Beck’s Depression Inventory, Insomnia Severity Index and Epworth Sleepiness Scale score

### Factors associated with CVD and hypertension in RLS

Subsequent analyses were performed only in patients with primary RLS to identify potential intrinsic risk factors of CVD or hypertension. CVD was reported by 106 (21.9%) and hypertension by 200 (41.4%) patients (Table 4). In univariate analysis, compared with patients without CVD, patients with RLS and CVD were significantly older, with hypercholesterolemia, sleep apnea and with older age at RLS and at daily RLS onset (Table 4). To identify which factors were independently associated with CVD, characteristics associated with CVD at p<0.15 were introduced in a multivariate model. Older age hypercholesterolemia and sleep apnea were significantly associated with CVD.

**Table 4:** Clinical, socio-demographic and restless legs syndrome (RLS) features in patients with primary RLS classified according to their cardiovascular disease (CVD) and hypertension status.

	CVD						Hypertension					
	No N = 377		Yes N = 106				No N = 283		Yes N = 200			
Variables	n	%	n	%	OR [95% CI]	p	n	%	n	%	OR [95% CI]	p
Sex (Women)	262	69.50	64	60.38	0.67 [0.43;1.05]	0.08	189	66.78	136	68.00	1.06 [0.72;1.56]	0.78
Age, years												
<61	88	23.34	7	6.60	1	0.0004	78	27.56	15	7.50	1	<0.0001
[61–69[	95	25.20	24	22.64	3.18 [1.30;7.74]		81	28.62	39	19.50	2.50 [1.28;4.90]	
[69–77[	96	25.46	29	27.36	3.80 [1.58;9.11]		61	21.55	64	32.00	5.46 [2.84;10.5]	
≥77	98	25.99	46	43.40	5.90 [2.53;13.7]		63	22.26	82	41.00	6.77 [3.56;12.9]	
Smoker status												
Non-smoker	267	71.20	66	62.26	1	0.13	180	64.06	153	76.50	1	0.006
Current smoker	27	7.20	7	6.60	1.05 [0.44;2.51]		27	9.61	7	3.50	0.31 [0.13;0.72]	
Former smoker	81	21.60	33	31.13	1.65 [1.01;2.68]		74	26.33	40	20.00	0.64 [0.41;0.99]	
Alcohol, g/day												
No Drink	116	31.69	23	22.55	1	0.14	86	31.16	53	27.60	1	0.70
≤12	190	51.91	55	53.92	1.46 [0.85;2.50]		144	52.17	102	53.13	1.15 [0.75;1.76]	
]12–24]	34	9.29	14	13.73	2.08 [0.96;4.47]		25	9.06	22	11.46	1.43 [0.73;2.78]	
]24–36]	11	3.01	7	6.86	3.21 [1.13;9.15]		9	3.26	9	4.69	1.62 [0.61;4.35]	
>36	15	4.10	3	2.94	1.01 [0.27;3.77]		12	4.35	6	3.13	0.81 [0.29;2.29]	
Physical activity												
No	86	22.93	30	28.57	1	0.24	52	18.51	64	32.16	1	0.0005
Yes, moderate	260	69.33	71	67.62	0.78 [0.48;1.28]		202	71.89	128	64.32	0.51 [0.34;0.79]	
Yes, intense	29	7.73	4	3.81	0.40 [0.13;1.22]		27	9.61	7	3.52	0.21 [0.08;0.52]	
Body Mass Index, kg/m2												
<25	187	50.13	43	41.35	1	0.27	159	56.58	70	35.71	1	<0.0001
[25–30]	132	35.39	42	40.38	1.38 [0.86;2.24]		87	30.96	88	44.90	2.30 [1.53;3.46]	
>30	54	14.48	19	18.27	1.53 [0.82;2.84]		35	12.46	38	19.39	2.47 [1.44;4.22]	
Cardiovascular family history (Yes)	115	31.08	41	40.20	1.49 [0.95;2.34]	0.08	102	36.56	111	57.51	2.35 [1.61;3.42]	<0.0001
Hypercholesterolemia (Yes)	72	19.41	45	43.69	3.22 [2.02;5.14]	<0.0001	41	14.64	75	38.86	3.70 [2.39;5.75]	<0.0001
Diabetes mellitus (Yes)	20	5.32	10	9.43	1.85 [0.84;4.09]	0.13	8	2.83	22	11.06	4.27 [1.86;9.80]	0.0006
Sleep apnea (Yes)	50	13.97	25	27.78	2.37 [1.37;4.10]	0.002	31	11.52	43	23.89	2.41 [1.45;4.00]	0.0007
Antidepressant intake (Yes)	48	12.90	18	17.14	1.40 [0.77;2.52]	0.27	39	13.93	26	13.27	0.95 [0.55;1.61]	0.84
Beck Depression Inventory												
No depression	154	44.38	43	45.26	1	0.98	123	47.31	75	41.21	1	0.16
Mild depression	105	30.26	27	28.42	0.92 [0.54;1.58]		78	30.00	54	29.67	1.14 [0.72;1.78]	
Moderate depression	57	16.43	17	17.89	1.07 [0.56;2.02]		43	16.54	31	17.03	1.18 [0.69;2.04]	
Severe depression	31	8.93	8	8.42	0.92 [0.40;2.16]		16	6.15	22	12.09	2.25 [1.11;4.56]	
Epworth Severity Scale (≥11)	163	46.05	41	43.16	0.89 [0.56;1.40]	0.62	120	44.61	85	46.96	1.10 [0.75;1.60]	0.62
Insomnia severity index												
No insomnia	27	7.46	7	7.00	1	0.93	20	7.41	15	7.77	1	0.18
Mild insomnia	107	29.56	32	32.00	1.15 [0.46;2.90]		89	32.96	51	26.42	0.76 [0.36;1.62]	
Moderate insomnia	177	48.90	49	49.00	1.07 [0.44;2.60]		132	48.89	94	48.70	0.95 [0.46;1.95]	
Severe insomnia	51	14.09	12	12.00	0.91 [0.32;2.57]		29	10.74	33	17.10	1.52 [0.66;3.50]	
Age at onset of 1^st^ RLS symptoms^(1)^ ^(2)^	42 [2–84]		50 [5–81]		1.13 [1.01;1.27]	0.04	40 [4–81]		47.5 [2–84]		1.15 [1.04;1.27]	0.006
Duration of RLS symptoms^(1)^	23.82 [0.15–86.12]		25.76 [2.15–80.22]		1.00 [0.99;1.02]	0.55	22.6 [2.20–86.12]		26.5 [0.15–79.86]		1.01 [1.00;1.02]	0.20
Age at onset of daily RLS^(1)^ ^(2)^	52 [2–90]		60 [15–82]		1.22 [1.03;1.43]	0.02	50 [8–85]		58.50 [2–90]		1.25 [1.09;1.43]	0.001
Duration of daily RLS^(1)^	13.82 [0.15–72.29]		13.62 [2.15–67.22]		1.01 [0.99;1.02]	0.36	13.14 [1.20–70.12]		14.82 [0.15–72.29]		1.01 [1.00;1.03]	0.08
Treatment for RLS (Yes)	342	90.72	99	93.40	1.45 [0.62;3.36]	0.39	252	89.05	189	94.50	2.11 [1.04;4.31]	0.04
Dopaminergic agonists (Yes)	285	75.60	85	80.19	1.31 [0.77;2.22]	0.32	212	74.91	158	79.00	1.26 [0.82;1.94]	0.30
Alpha2/delta ligands (Yes)	49	13.00	13	12.26	0.94 [0.49;1.80]	0.84	30	10.60	31	15.50	1.55 [0.90;2.65]	0.11
RLS severity						0.47						0.61
Mild/Moderate	69	19.77	19	20.65	1		53	20.31	35	19.44	1	
Severe	208	59.60	49	53.26	0.86 [0.47;1.55]		156	59.77	102	56.67	0.99 [0.60;1.62]	
Very severe	72	20.63	24	26.09	1.21 [0.61;2.41]		52	19.92	43	23.89	1.25 [0.70;2.25]	
RLS family history (Yes)	177	47.97	42	42.86	0.81 [0.52;1.27]	0.37	132	48.35	85	43.81	0.83 [0.58;1.21]	0.33
RLS upper limb (Yes)	170	46.20	53	51.96	1.26 [0.81;1.95]	0.30	124	45.42	100	50.76	1.24 [0.86;1.79]	0.25
Ferritin, ng/ml (≥50)	124	92.54	40	86.96	0.54 [0.18;1.57]	0.26	89	87.25	74	94.87	2.70 [0.85;8.64]	0.09
RLS augmentation												
No	202	60.48	55	57.89	1	0.88	148	60.41	108	58.70	1	0.19
Probable	100	29.94	31	32.63	1.14 [0.69;1.88]		79	32.24	53	28.80	0.92 [0.60;1.41]	
Certain	32	9.58	9	9.47	1.03 [0.47;2.29]		18	7.35	23	12.50	1.75 [0.90;3.40]	
RLS Quality of life–Global score^(1)^	33 [3–60]		30 [6–54]		0.99 [0.97;1.01]	0.23	32.5 [4–60]		33 [3–58]		1.00 [0.99;1.02]	0.59

^(1)^ Quantitative variables are expressed as median [minimum value-maximum value]

(2) OR for 10 year-increase

Among the 106 patients with RLS and CVD, 74.5% (n = 73) reported the occurrence of CVD after RLS onset. In comparison with patients without CVD, these 73 patients were significantly older and had more often hypertension, hypercholesterolemia, sleep apnea and family history of CVD. In univariate analysis, compared with patients with RLS but without hypertension, patients with hypertension were significantly older and more overweight/obese with less physical activity and reported less tobacco consumption, more hypercholesterolemia, diabetes mellitus, sleep apnea, family history of CVD, older age at RLS and daily RLS onset and took more drugs for RLS (Table 4). Multivariate model including factors associated with hypertension at p<0.15 reported that older age, a lower tobacco consumption, an overweight, a family history of CVD, hypercholesterolemia, diabetes and sleep apnea were independently associated with hypertension.

In patients with RLS and CVD or/and hypertension, no significant association was found with RLS severity, RLS-related quality of life, RLS duration, family RLS history, ferritin levels, presence of RLS symptoms in arms and presumed augmentation syndrome. Moreover, no between-group difference was found concerning the presence of EDS, insomnia and depressive symptoms (Table 4).

## Discussion

In this case-control study, we reported that most of treated patients with primary RLS had significantly higher frequency of CVD and hypertension than controls in unadjusted analysis; however, this association was no longer significant after adjustment for socio-demographic and metabolic characteristics, depressive and sleep symptoms. Among patients with RLS, those with CVD or hypertension reported older ages at first RLS and at daily RLS symptom onset when compared to patients without CVD or hypertension. Conversely, no relationship was found between CVD and hypertension and RLS severity, duration, augmentation, daytime sleepiness, insomnia and depressive symptoms.

RLS patients often have high cardiovascular risk factors, such as advanced age, obesity [4], diabetes mellitus and hypercholesterolemia [3], as confirmed by our study. Sleep disturbances, insomnia, EDS and depressive symptoms, which increase the risk of CVD and hypertension [24,25,26] are also frequently associated with RLS [22,23], a finding also confirmed by the present study. PLMs are strongly associated with RLS, concomitantly with sleep fragmentation and recurrent increases in blood pressure and heart rate that may trigger a non-dipper pattern of blood pressure (i.e. defined as a nocturnal systolic or diastolic blood pressure decrease lower than 10% of the daytime blood pressure). [8,33]. This could promote sympathetic arousal, activate the hypothalamic pituitary adrenal axis and increase the levels of pro-inflammatory cytokines and circulating catecholamine, all conditions that favor CVD and hypertension. Moreover, the genetic RLS background could also increase CVD risk. For instance, a recent study reported the alteration of the sympatho-vagal regulation of cardiac rhythmicity in mice upon inactivation of Meis 1, one of the most important RLS susceptibility loci [11,34,35].

The two largest cross sectional studies showed significant association between RLS and CVD [13,19]. However, longitudinal population-based studies more prompt to demonstrate a causal relationship between RLS and CVD and/or hypertension showed inconsistent results [10,12–21]. Although large size samples were included in some studies, they mostly focused on the general population. Therefore, and differently from our study, they could have included people with mild RLS disease who never consulted a physician and who did not take any RLS medication. In some studies reporting higher frequency of CVD or hypertension in RLS, the possibility remains that unmeasured confounders explain part of the reported association [23,36]. In contrast, other studies that adjusted for age and sex, and other potential confounders (e.g., BMI, cholesterol, physical activity…) were less likely to find an association between CVD, hypertension and RLS. A recent large retrospective cohort study reported that primary RLS was associated with an increased risk of hypertension, but not with CVD, whereas secondary RLS was associated with both [18]. However, this double association in secondary RLS could have been related to the underlying disorder that might increase CVD risk (e.g., a renal disease) and not to RLS per se. Moreover, this study did not include information on RLS severity, concomitant sleep disturbances and RLS treatment intake.

Here, we included AFE members (an RLS Patients Association) having the four cardinal RLS criteria. All included patients had a disease severe enough to require medical care (severe to very severe condition in 80% of patients at the time of study, despite drug treatment in 91.2% of them). We found that 21.9% of patients with primary RLS had CVD and 41.4% hypertension. These frequencies were significantly higher than among controls in unadjusted associations, but not after adjustment for confounding factors, such as demographic and metabolic variables, sleep and depressive symptoms. We also investigated potential intrinsic risk factors for CVD and hypertension in patients with RLS and found that patients with CVD/hypertension were significantly older and had hypercholesterolemia, sleep apnea, but also older age at RLS and daily RLS onset. Conversely, RLS duration, severity, family history, presumed augmentation syndrome, low ferritin, daytime sleepiness, depressive and insomnia symptoms were not associated with CVD or hypertension.

RLS medications also could have affected the frequency of CVD and hypertension. Although 91.2% of patients were taking an RLS treatment, we found that RLS treatment was associated with hypertension, but not CVD. This suggests that patients with severe RLS that required medication may be at greater risk for hypertension. On the other hand, RLS drugs, especially dopaminergic agonists (taken by 84% of patients), may protect these patients from hypertension and CVD by decreasing sleep fragmentation and PLM. For instance, a recent double-blind, placebo-controlled study reported that rotigotine, a dopaminergic agonist, reduced the PLM-associated systolic blood pressure elevations in patients with primary RLS [33]. Unfortunately, the number of untreated patients with RLS in our study was insufficient to perform a sensitivity analysis to compare RLS treatment/absence of treatment on the association between RLS and CVD and hypertension.

The present study has some limitations. Almost all patients with RLS were treated with drugs (mostly a dopaminergic agonist) and this could have affected the association between RLS and CVD/hypertension. The recruitment of participants from an RLS Patients Association with absence of face-to-face interview may have exposed to potential selection bias and may not well represent RLS patients in the general population and in tertiary centers. Only a subgroup of patients from the RLS Patient Association participated in this study (i.e. responders) with potential demographic and clinical differences with the non-responders. Indeed, patients with more severe disease or more comorbid conditions could have been more motivated in participating and, thus, our population might not accurately reflect the French RLS population. However, cases and controls received similar instructions, answered the questionnaire on a voluntary basis and were not randomly selected. Patients with secondary RLS condition were excluded due to a small number of subjects, despite a potentially high risk of CVD and hypertension incidence reported in this population [18]. In most cases, the controls were the spouses of the patients that explain the gender difference between the populations included, with potential bias when considering the high CVD risk factor in men, more numerous in the control group. CVD outcomes were assessed using the self-administered questionnaire that relies on the memory of subjects and thus may have contribute to recall bias. The absence of significant association between CVD /hypertension and RLS after adjustments for covariates (including at least gender, age and BMI) should be interpreted with caution regarding the self-reported CVD outcomes and the small number of events per exposure variable. Finally, there were no polysomnographic recordings quantifying sleep fragmentation and PLM.

The present study has several strengths. This case-control study had a suitable design to study disease with low prevalence even if it is difficult to establish a directional link between RLS, CVD and hypertension. All patients completed a questionnaire that fully assessed RLS severity, duration, age at onset, family history, medication intake, presumed RLS augmentation, sleep-associated problems and depressive symptoms. We focused on primary RLS to avoid heterogeneous comorbid conditions that may explain the association with CVD or hypertension independently of RLS. Different CVD events were identified and defined according to standardized criteria to minimize the classification bias. We adjusted our results in patients and controls for a wide range of potential confounders, including socio-demographic and lifestyle factors, established cardiovascular risk factors, chronic disorders, psychological distress and sleep complaints.

To conclude, despite some limitations in the design of this study that relies on self-reported CVD and hypertension outcomes, we did not find any significant association with primary RLS after controlling for a large number of potential confounders. Patients with RLS, CVD or hypertension had higher established cardiovascular risk factors, older ages at first RLS and at daily RLS symptom onset than patients without CVD or hypertension. As almost all patients received RLS-related treatment, mostly a dopaminergic agonist, these drugs could have prevented hypertension and CVD and thus led to underestimate this association. Further prospective studies to investigate CVD proxy (e.g., ambulatory blood pressure monitoring and endothelial function) instead of CVD are required to better understand the impact of RLS on cardiovascular risk and the potential changes linked to RLS treatment.
